# Astrocytic GPR55 receptors promote glycolysis

**DOI:** 10.1186/s42238-026-00407-x

**Published:** 2026-04-09

**Authors:** Cândida Dias, Erik Keimpema, Rui A. Carvalho, Daniela Madeira, Liliana Dias, Ana Ledo, João Laranjinha, Rodrigo A. Cunha, Paula Agostinho, Tibor Harkany, Attila Köfalvi

**Affiliations:** 1 CNC-UC - Center for Neuroscience and Cell Biology, University of Coimbra, Coimbra, 3004-504 Portugal; 2 Faculty of Pharmacy, University of Coimbra, Coimbra, 3000-548 Portugal; 3 Department of Molecular Neurosciences, Center for Brain Research, Medical University of Vienna, Vienna, 1090 Austria; 4 Life Sciences Department, Faculty of Sciences and Technology, University of Coimbra, Coimbra, 3000-456 Portugal; 5 Associated Laboratory for Green Chemistry (LAQV), Group of Pharmaceutical Technology, Faculty of Pharmacy, REQUIMTE, University of Coimbra, Coimbra, 3000-548 Portugal; 6 Faculty of Medicine, University of Coimbra, Coimbra, 3004-504 Portugal; 7 Department of Neuroscience, Karolinska Institutet, Biomedicum 7D, Solna, 17165 Sweden; 8 CiBB - Centre for Innovative Biomedicine and Biotechnology, University of Coimbra, Coimbra, 3000-548 Portugal

**Keywords:** Astrocyte, Cannabinoid, Cortex, Glucose uptake, GPR55, Cellular respiration, Hippocampus, Lactate, Mitochondria, NMR

## Abstract

**Background:**

The endocannabinoid system is closely associated with systemic and cellular energy metabolism in mammals. We previously demonstrated that the cannabinoid receptors CB_1_ and CB_2_ play important roles in cerebral glucose metabolism. GPR55, a paracannabinoid receptor, has been implicated in systemic metabolic regulation and in diseases such as intractable epilepsy, diabetes, and cancer. In the present study, we investigated whether GPR55 also influences brain glucose metabolism.

**Methods:**

Acute hippocampal and cortical slices and primary astrocytic cultures from C57BL/6 mice and Wistar rats were used. Quantitative polymerase chain reaction (qPCR) was used to assess *Cnr1* and *Gpr55* gene expression levels, and [³H]deoxyglucose/[¹⁴C]-U-glucose uptake assays, high-resolution respirometry using the Oroboros O2k system, and proton nuclear magnetic resonance ([^¹^H]-NMR) spectroscopy were employed to assess metabolic activity upon receptor activation.

**Results:**

qPCR analysis revealed that *Cnr1* mRNA dominated in neurons, whereas *Gpr55* was predominantly expressed in primary astrocytes. Δ⁹-Tetrahydrocannabinol (Δ⁹-THC), the GPR55-selective synthetic agonist O-1602, the endogenous GPR55 agonists, L-α-lysophosphatidylinositol (LPI) and palmitoylethanolamide (PEA), together with the mixed CB_1_ receptor antagonist/GPR55 agonist AM251, invariably and significantly stimulated glucose uptake and metabolism in brain preparations at nanomolar concentrations ex vivo. The GPR55-selective antagonist CID16020046 (1 µM) abolished the effects of the GPR55 agonists, as did pretreatment with the cytosolic Ca^2+^ chelator BAPTA-AM (30 µM). While LPI did not alter oxidative metabolism in either astrocytes or neurons, it selectively stimulated glycolysis and lactate release in astrocytes.

**Conclusions:**

Our findings reveal a novel role for GPR55 in astrocytes. By enhancing glycolytic activity in these cells, GPR55 is likely poised to support, even if indirectly, the energy demands of synaptic transmission.

**Supplementary Information:**

The online version contains supplementary material available at 10.1186/s42238-026-00407-x.

## Introduction

Cannabis research took its first major leap during the hippie era, when Gaoni and Mechoulam determined the chemical structures of the two principal phytocannabinoids, cannabidiol (CBD) and Δ⁹-tetrahydrocannabinol (Δ⁹-THC) (Mechoulam et al. 2014). Δ⁹-THC interacts with a variety of receptors throughout the body, but only the CB_1_ and CB_2_ cannabinoid receptors (CB_1_Rs and CB_2_Rs) are considered canonical components of the endocannabinoid system (ECS) (Solymosi and Köfalvi, 2017). The best-characterized endogenous ligands for CB_1_R and CB_2_R are *N*-arachidonoylethanolamine (anandamide) and 2-arachidonoylglycerol (2-AG).

Beyond CB_1_R and CB_2_R, a third G protein-coupled receptor (GPCR) – the former orphan receptor GPR55 (Sawzdargo et al. 1999) – is activated by Δ⁹-THC, anandamide, 2-AG, and certain synthetic ligands, including O-1602 and AM251 (Ryberg et al. 2007; Anavi-Goffer et al. 2012). Notably, AM251 was originally considered a highly selective CB_1_R antagonist/inverse agonist (Pertwee 2005). Interestingly, a number of endogenous lipids that lack significant activity at CB_1_R and CB_2_R can stimulate GPR55-mediated signaling instead. Among them, palmitoylethanolamide (PEA), a fatty acid amide with well-documented central effects (e.g. Kramar et al. 2017; Patricio et al. 2022), is an efficacious, even if non-selective agonist. Meanwhile, L-α-lysophosphatidylinositol (LPI) (Oka et al. 2007), 2-arachidonoyl-sn-glycero-3-phosphoinositol (Oka et al. 2009) and lyso-phosphatidyl-β-d-glucoside (Guy et al. 2015) have been identified as potent and selective endogenous GPR55 agonists. In contrast, CBD mostly acts as an antagonist/inverse agonist at the GPR55 (Ryberg et al. 2007; Anavi-Goffer et al. 2012; Patricio et al. 2022), at least at micromolar concentrations.

The widespread expression of endocannabinoid receptors, ligands, and the associated enzymatic machinery indicates that the ECS plays fundamental roles in mammalian (patho)physiology (Ligresti et al. 2016; Lowe et al. 2021; Maccarrone et al. 2023; Simankowicz and Stępniewska 2025). Among these functions, the regulation of energy metabolism at systemic, central, and cellular levels (Matias et al. 2008; Piazza et al. 2017) is particularly relevant to the current study. For instance, it is acknowledged that both acute and chronic exposure to Δ⁹-THC affect systemic glucose metabolism and insulin sensitivity (de Pasquale et al. 1978; Matias et al. 2008; Penner et al. 2013). The potent glucoregulatory effects of the ECS are highly relevant to metabolic and neuropsychiatric disorders as well as cancer (Simankowicz and Stępniewska 2025).

We and others have shown that much of the cannabinoid impact on systemic glucose homeostasis is mediated through CB_1_Rs in the pancreas (Bermúdez-Siva et al. 2006, 2007; Malenczyk et al. 2013, 2015). Some of these effects may also stem from physical interactions between CB_1_Rs and insulin or insulin-like growth factor-1 receptors, where CB_1_R activation inhibits downstream signaling (Dalton and Howlett 2012). As for CB_2_Rs, systemic administration of the CB_2_R-selective agonist JWH133 (Huffman et al. 1999) was shown to improve, while CB_2_R blockade impaired, glucose tolerance (Bermúdez-Silva et al. 2007). Because CB_2_R activation appears to suppress glucose-dependent insulin secretion (Bermúdez-Silva et al. 2008), the improved glucose clearance observed after JWH133 treatment may reflect CB_2_R-mediated enhancement of cellular glucose uptake.

The role of GPR55 in systemic glucose metabolism has also been highlighted (Tudurí et al. 2017). For example, GPR55 activation in pancreatic β-cells stimulates insulin release via IP_3_-mediated increases in cytosolic Ca^2+^ (Vong et al., 2019), and an overactive GPR55–LPI axis has been associated with obesity and enhanced lipogenesis in white adipose tissue (Moreno-Navarrete et al. 2012). Moreover, *Gpr55* deletion in obese mice reduces glucose tolerance and, intriguingly, diminishes the effects of cannabinoids on body weight (Wargent et al. 2020; Liu et al. 2024). Finally, functional polymorphisms in *GPR55* have been linked to anorexia nervosa in Japanese women (Ishiguro et al. 2011).

While investigating the role of the ECS in brain energy metabolism (Lemos et al. 2012; Köfalvi et al. 2016; Moura et al. 2019; Pedro et al. 2020), we observed that both Δ⁹-THC – a partial agonist at CB_1_R and CB_2_R – and AM251 – a CB_1_R-selective antagonist/inverse agonist (Pertwee 2005) – stimulated glucose uptake in acute brain slices and astrocyte cultures. These unpublished observations could be best explained by a receptor mechanism involving GPR55 rather than CB_1_R or CB_2_R. GPR55 has already been implicated in intractable epilepsy (Kaplan et al. 2017; Gray and Whalley 2020), and in supporting glycolysis in pancreatic cancer (Bernier et al. 2017). The hypothesis that GPR55 sustains heightened metabolic states (in development, adult physiology, and disease) through increasing aerobic glucose metabolism prompted us to study its role in brain glucose metabolism and cellular respiration.

## Materials and methods

### Animals

Colonies of Wistar rats and C57BL/6 mice were maintained at the local animal facility. We have used 6–10 week-old male rats and mice for the study. All animals were housed on a 12 h light/dark cycle under controlled temperature conditions (23 ± 2 °C), with *ad libitum* access to food and water, and were supervised by the attending veterinarian. Every effort was made to minimize the number of animals used and to reduce their stress and discomfort. Prior to the in vitro experiments, adult rodents were deeply anesthetized with halothane (5%, 1 L/min flow rate), confirmed by the absence of a response to tail pinch or handling, while spontaneous breathing was maintained.

### Glucose uptake in brain slices

The following in vitro experiments in rat and mouse brain slices were optimized over the past 15 years and performed with slight modifications from our previous protocols (Lemos et al. 2012, 2015; Köfalvi et al. 2016). Anesthetized animals were decapitated (at approximately 14:00 h each experimental day to minimize potential circadian hormonal effects), and their brains were immediately collected in ice-cold assay solution (see below). After cooling, hippocampi and neocortices were removed on ice within 4 min post-decapitation and sliced into 450 μm-thick transverse sections using a McIlwain tissue chopper. Slices were gently separated in ice-cold modified Krebs–HEPES assay solution (aCSF) containing (in mM): NaCl 132, KCl 3, KH_2_PO_4_ 1.2, MgSO_4_ 1.2, CaCl_2_, 2.5, NaHCO_3_ 25, glucose 5.5, and HEPES 10, under gentle oxygenation with 95% O₂ and 5% CO₂ (i.e. carbogen) (pH, 7.4).

Five hippocampal or three frontal cortical slices were then transferred into a multi-chamber slice incubator containing 50 mL assay solution at 37 °C and continuously bubbled with carbogen until the end of the experiment, as before (Lemos et al. 2012, 2015; Köfalvi et al. 2016; Moura et al. 2019; Pedro et al. 2020). After a 60-minute pre-incubation period, which was necessary to reach steady-state glucose metabolism ex vivo (Lemos et al. 2012) – vehicle (DMSO, 0.1% v/v), various concentrations of cannabinoids, or KCl were applied (NaCl served as the osmotic control for KCl). In all slice-based experiments, multiple hippocampal slices were obtained from each animal, but all slices from a given animal were pooled and counted as a single biological replicate (“n”).

When the antagonist effect of CBD (1 µM) and CID16020046 (1 µM) was tested, each was added 5 min prior to the cannabinoid agonists. When the role of cytosolic Ca^2+^ elevation was tested, BAPTA-AM (30 µM) was applied during the first 40 min of the recovery period, after which slices were transferred to new chambers containing fresh, BAPTA-free Krebs solution. Importantly, the concentration of BAPTA-AM was chosen to be slightly above its EC_50_ in rat cells (Tojyo and Matsumoto 1990) to avoid potential toxicity and metabolic perturbations. For BAPTA control conditions, slices were exposed to DMSO alone for 40 min and then transferred to DMSO-free assay medium.

Following the 5-minute incubation with cannabinoid ligands or immediately after KCl application, 2-[^3^H]-deoxy-D-glucose ([³H]DG; final cc.: 2.5 nM; specific activity: 60 Ci/mmol; American Radiolabeled Chemicals, Inc. [ARC], Saint Louis, MO, USA) was bath-applied. To quantify non-specific external [^3^H] labeling, an independent mock control was performed on ice – a condition known to halt membrane transport processes (Sumiya et al. 2001), enabling measurement of non-specific [^3^H]DG surface binding. After a 30-minute uptake period, slices were washed twice in ice-cold uptake solution (5 min each) and collected in 1 mL of 0.5 M NaOH. From each 1 mL sample, 800 µL were assayed for [^3^H] counts using a Tricarb β-counter (PerkinElmer, USA), while the remaining volume was used to determine protein concentration using the bicinchoninic acid (BCA) assay, also known as the Smith assay (Smith et al. 1985; Merck Biosciences, Germany).

The incubation bath was also sampled and assayed for [^3^H] content, allowing calculation of the total nanomoles of glucose (cold + radiolabeled) corresponding to the actual radioactivity retained in the slices. After subtracting the non-specific [^3^H] signal from each total uptake value, the resulting specific uptake was expressed as nmol/mg protein. We refer to Lemos et al. (2012) for further details regarding optimization steps, calculation procedures and formulae.

### High-resolution respirometry

Experiments were carried out as previously described (Dias et al. 2025a). Hippocampi from 11 rats were dissected, and 400 μm slices were cut using a Vibroslice (World Precision Instruments, Inc.). The chamber of the Vibroslice was filled with ice-cold modified aCSF and continuously bubbled with carbogen during slice preparation. Next, slices were placed in a pre-incubation chamber (BSC-PC, Harvard Apparatus) containing modified aCSF at room temperature that had been continuously bubbled with carbogen. Slices were allowed to recover for at least 1 h under these conditions. Tissue oxygen consumption rate was measured by high-resolution respirometry with the help of an Oxygraph-2k (Oroboros Instruments, Innsbruck, Austria) as follows:

Measurements were carried out with continuous stirring (750 rpm) in 2 mL of Krebs medium at 37 °C and at high oxygen concentrations (approximately 650 to 850 µM) to ensure proper oxygenation of the entire tissue. Calibration was performed at air concentration according to the manufacturer’s instructions. A manufactured slice holder was placed in the chamber to prevent mechanical damage from stirring, and two slices were used per chamber (average wet tissue weight of 3.8 mg). After establishing a stable basal oxygen flux (for details, see Dias et al. 2025a) LPI (100 nM) or its vehicle, DMSO (0.1% v/v), was added to the chamber for a minimum period of 30 min (see Fig. 3A). Subsequently, fluorocitrate (final concentration: 100 µM) was added into the recording chamber for a minimum period of 1.5 h to impair carbon flux through the Krebs cycle (Swanson and Graham 1994; Fig. 3A, B).

To prepare the fluorocitrate solution, we followed the published protocol (Swanson and Graham 1994; Dias et al. 2023): First, 8 mg of DL-fluorocitric acid barium salt (Sigma) were dissolved in 105 µL of 0.57 M HCl; then barium ions were precipitated with 43 µL of 0.7 M Na_2_SO_4_, followed by the addition of 32 µL of 0.94 M NaCO_3_. The suspension was centrifuged at 1000 *g* for 5 min to recover the supernatant containing fluorocitrate.

### Astrocyte cultures for metabolic assays

Primary astrocyte cultures were prepared from the cerebral cortices of postnatal day 1 to 3 Wistar rats, as previously described (Dias et al. 2025b). After sacrificing the female Wistar dams, each litter (typically a pool of 8 to 10 pups) was used for a single cell culture preparation. Following removal of the meninges, the cortices were mechanically and enzymatically dissociated using a scalpel and TrypLE reagent (Gibco), supplemented with DNase I (10 mg/mL in 10 mM NaCl, Sigma-Aldrich). Enzymatic digestion was halted by adding culture medium consisting of high-glucose Dulbecco’s Modified Eagle Medium, supplemented with 10% fetal bovine serum and 10 mL/L penicillin–streptomycin, adjusted to pH 7.4.

The cell suspension was centrifuged at 115 *g* for 2 minutes, with their pellet resuspended in fresh culture medium. Cells were then seeded in T75 flasks pre-coated with poly-D-lysine (0.1 mg/mL in borate buffer, pH 8.2) at a density of 1 × 10^5^ cells/cm^2^ and maintained in a 5% CO_2_ incubator at 37 °C until reaching confluence. The culture medium was replaced every 2 to 3 days. Microglia were removed from the astrocyte monolayer by shaking at 200 rpm for 2 hours at 37 °C, followed by complete medium replacement, resulting in an astrocyte culture with ~98% purity (Dias et al., 2025b).

Two to three days after re-establishing cultures, astrocytes were detached using a mild trypsinization protocol. Cells were first washed with phosphate-buffered saline (PBS: 135 mM NaCl, 2.7 mM KCl, 4.3 mM Na_2_HPO_4_, and 1.47 mM KH_2_PO_4_) containing 1 mM EDTA, then detached using PBS with 0.05% trypsin (Sigma-Aldrich). The cell suspension was centrifuged at 180 *g* for 5 min, and the astrocytes were reseeded in 24-well multiplates at a density of approximately 150,000 cells/cm^2^ and maintained until confluence. After two days of readjustment, the cultures were used for glucose uptake experiments.

### Metabolic assays in astrocyte cultures

The tandem [^3^H]DG/[^14^C]_6_-glucose uptake assay was performed as previously described (Lemos et al. 2012, 2015; Köfalvi et al. 2016), with slight modifications. The rationale behind this technique is that [^3^H]DG, a glucose analog, is generally assumed to represent net glucose uptake, as it becomes phosphorylated upon cellular entry and thus trapped within the cells. In contrast, [^14^C]_6_-U-glucose contains six [^14^C]-labelled carbon atoms that can engage in metabolic processes and subsequently leave the cells as either [^14^C]CO_2_ or lactate.

This method, pioneered by our group, allows the calculation of glucose uptake per mg of protein based on [^3^H] counts, and the determination of glucose-derived carbon retention based on [^14^C] counts. Therefore, multiplying the difference between final [^3^H] and [^14^C] contents (both expressed as nmol/mg protein) by six provides a realistic measure of how many nmol/mg protein of carbon atoms were lost through dissipative metabolism (Lemos et al. 2012).

For the assay, the culture medium was replaced with assay medium similar to that used for brain slices, except with its D-glucose concentration being 3 mM. After preincubating for 1 h at 37 °C, test compounds or vehicle (maximum DMSO concentration: 0.1% v/v) were added to each well. Five minutes later, [³H]DG (for specifications, see above; final cc.: 5 nM) and [^14^C]_6_-(uniformly labeled)-D-glucose (final cc.: 1.5 µM; specific activity: 360 mCi/mM; Perkin Elmer, USA) were added to the medium, in a total incubation volume of 300 µL.

A 100 µL aliquot of the assay medium was also collected to relate [^3^H] and [^14^C] counts to 0.003 mol/L × 0.0001 L of glucose, corresponding to the amount of cold glucose in that volume. Following the 30-minute incubation, wells were thoroughly but gently washed with ice-cold assay medium. The remaining cellular content was then lysed using 0.5 M NaOH and used for dual-label β-counting and protein quantification.

### [^1^H]-NMR spectroscopy

The treatment of *n* = 5 independent astrocyte cultures with 100 nM LPI was carried out similarly to the assay above, yet without the radioactive tracers and in a volume of 500 µL. After 30 min of incubation with LPI or DMSO at 37 °C, the NMR spectra of the collected 500 µL culture medium were acquired as previously reported (Alves et al. 2011). Acquisition was done at 14.1 T, 25 °C, using a Varian 600-MHz spectrometer equipped with a 3-mm indirect detection probe with a z-gradient (Varian Instruments, Palo Alto, CA, USA). Solvent-suppressed [^1^H]-NMR spectra were acquired with a sweep width of 6 kHz, using a delay of 14 s to allow total proton relaxation, a water pre-saturation of 3 s, a pulse angle of 45°, an acquisition time of 3.5 s and at least 64 scans were registered. The relative areas of [^1^H] doublets were quantified using the curve-fitting routine supplied with the NUTSproTM NMR spectral analysis program (Acorn, NMR Inc., Fremont, CA, USA). The concentrations of the metabolites present in the [^1^H]-NMR spectra obtained for the perfusates were calculated using sodium fumarate (2 mM) as standard internal reference.

### Quantitative polymerase chain reaction (qPCR)

Primary cultures from E16.5 mouse or P1 rat cortices were established and maintained for 4 days at a density of 1 × 10^6^ cells per well in poly-D-lysine-coated six-well plates (*n* = 3). Cortical homogenates were obtained from 2 month-old rats. A qPCR protocol was carried out based on Obara et al. (2011), with slight modifications: RNA was extracted using the RNeasy mini kit (Qiagen) with a DNase I digestion also performed to eliminate traces of genomic DNA and reverse transcribed using a high-capacity cDNA reverse transcription kit (Applied Biosystems). qPCR was performed on MyIQ instrument (BioRad) using iQ SYBR Green Supermix.

Samples were tested in triplicates using the following primer pairs: *Gpr55* (mouse-forward), 5′-GTCCATATCCCCACCTTCCT-3’; *Gpr55* (mouse-reverse), 5′-CATCTTGAATGGGAGGGAGA-3’; *Gpr55* (rat-forward), 5′-CTCCCTCCCATTCAAGATGA-3’; *Gpr55* (rat-reverse), 5′-AAGATCTCCAGGGGGAAGAA-3’ (Obara et al. 2011); *Cnr1* (forward), 5′-TCTTAGACGGCCTTGCAGAT-3’; *Cnr1* (reverse), 5′-AGGGACTACCCCTGAAGGAA-3’; mouse glyceraldehyde-3-phosphate dehydrogenase (*Gapdh*) (forward), 5′-AACTTTGGCATTGTGGAAGG-3’ and *Gapdh* (reverse), 5′-ACACATTGGGGGTAGGAACA-3’; rat *Gapdh* (forward): 5′-CAAGTTCAACGGCACAGTCA-3’ and rat *Gapdh* (reverse): 5′-CCCCATTTGATGTTAGCGGG-3’. Species-specific primer pairs were used for mouse and rat samples, and amplicon specificity and expected product size were verified by agarose gel electrophoresis.

Samples were amplified after an initial denaturation stage of 10 min at 95 °C, followed by 40 cycles of denaturation at 95 °C for 15 s and annealing and extension for 1 min at 60 °C. An additional dissociation curve step (from 60 to 95 °C with 0.5 °C steps for 10 s each) was performed to confirm the absence of non-specific products. Samples without template served as negative control. Expression levels were obtained by normalizing to the housekeeping gene encoding *Gapdh* obtained for every sample in parallel assays. Relative expression was calculated using the ΔΔCq (ΔΔCt) method as implemented in the Bio-Rad CFX analysis software, with *Gapdh* serving as the internal reference. For full uncropped gels and further explanations, see Supplementary Fig. 1.

### Chemicals used

Carbogen gas mixture (O_2_/CO_2_ 95%/5%) was obtained from Linde, Portugal. All water-based solutions were prepared in bi-deionized MilliQ water with resistivity ≥ 18 MΩ × cm (Millipore Corporation, USA). AM251 and Δ^9^-tetrahydrocannabinol (THC) were purchased from Abcam Biochemicals, Cambridge, UK. BAPTA-AM, (-)-cannabidiol (CBD), CP945598, JWH133, LY320135, O-1602, O-2050, and PF514273 were bought from Tocris Bioscience, Bristol, UK. CID16020046, L-α-lysophosphatidylinositol sodium (LPI), palmitoylethanolamide (PEA), HEPES, sucrose, DL-fluorocitric acid barium salt, DMSO and all other chemicals were analytical-grade and purchased from Sigma-Aldrich/ MerckBiosciences (Darmstadt, Germany). The receptor ligands were aliquoted and frozen at various concentrations to always allow their use at a 1:1000 dilution (final DMSO concentration, 0.1%).

### Data treatment

All data represent means ± SEM of *n* ≥ 6 observations (6 animals) except where noted. For the glucose uptake study, raw data were normalized to the vehicle control of the same experiment. All statistical analyses were carried out with the help of GraphPad Prism 8.02. Normalized data were tested for normality by the D’Agostino and Pearson omnibus normality tests. Statistical significance was calculated by one-sample *t-*test, and when more than one tests disagreed, Wilcoxon signed rank test was performed on data medians against a hypothetical control value. All other data were evaluated by paired *t*-test or repeated measures ANOVA with Dunnett’s *post-hoc* or two-way ANOVA followed by Sidak *post-hoc* test with correction for multiple comparisons, as indicated in the text. A p value of < 0.05 was considered significant.

## Results

### GPR55 agonists stimulated glucose uptake in acute brain slices

The majority of the pharmacological experiments was carried out in rat hippocampal slices, as we have previously characterized this technique in detail (Lemos et al. 2012; Pedro et al. 2020), and it has proven to be the fastest tool for conducting robust pharmacological analyses. Glucose uptake amounted to 67.5 ± 1.7 nmol/mg protein in the control hippocampal slices of the *n* = 122 rats used for this assay. In cortical slices from 8 of these rats, DMSO control glucose uptake amounted to 61.4 ± 2.3 nmol/mg protein. Finally, glucose uptake in cortical slices from 6 mice was 57.0 ± 3.6 nmol/mg protein.

**Fig. 1:**
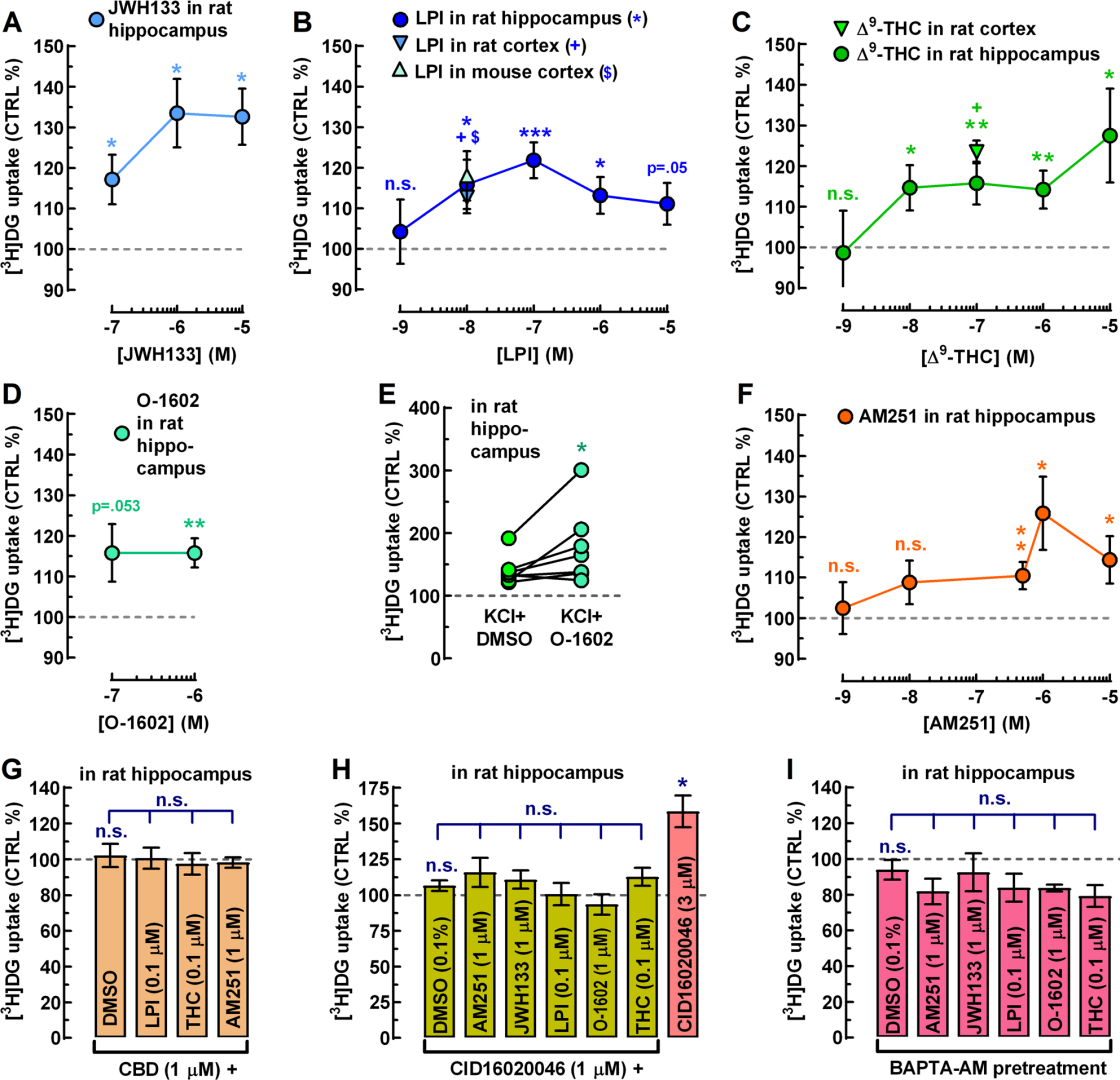
Nanomolar concentrations of GPR55 agonists stimulate glucose uptake in acute rat and mouse brain slices. Glucose uptake was quantified by [^3^H]DG incorporation, using a protocol optimized for rat hippocampal slices (Lemos et al. 2012). Rat and mouse cortical slices complemented experiments conducted in cortical neurons and astrocytes from both species (see Figs. 2, 4 and 5). **A** The cannabinoid CB_2_R-selective agonist JWH133 stimulates glucose uptake in a concentration-dependent fashion (*n* = 5–6 independent observations, i.e. rats), serving as a positive control (as per Köfalvi et al. 2016). **B** The endogenous GPR55 agonist, LPI, significantly stimulated glucose uptake in the rat hippocampus starting at 10 nM (*n* = 11), with maximal effect at 100 nM (*n* = 25). At 10 µM, LPI’s effect was only borderline significant (*n* = 12). LPI (10 nM) also increased glucose uptake in rat (*n* = 8) and mouse (*n* = 6) cortical slices, to a similar extent as in the rat hippocampus. **C** Δ⁹-THC, the principal psychoactive component of cannabis, significantly stimulated glucose uptake in (*n* ≥ 6) rat hippocampal and cortical slices (*n* = 6), displaying a non-monotonic concentration–response profile, with significant stimulation of glucose uptake apparent from 10 nM (*n* = 9). **D** The GPR55-selective synthetic agonist O-1602 significantly stimulated glucose uptake at 1 µM in the rat hippocampus (*n* = 10). **E** O-1602 (1 µM) enhanced glucose uptake during 20 mM KCl-induced depolarization (*n* = 7 rats), indicating that GPR55 promotes glucose uptake under both resting and depolarized states. **F** AM251 showed lower potency than Δ^9^-THC and LPI, with a minimum effective concentration of 500 nM (*n* = 41), consistent with its lower GPR55 vs. CB_1_R affinity (Ryberg et al. 2007). **G** Pretreatment with CBD (1 µM) fully prevented the stimulatory effects of LPI (100 nM), Δ^9^-THC (100 nM), and AM251 (1 µM) (*n* = 6 each). Note that the leftmost bar shows the antagonist control condition (CBD + DMSO, where DMSO stands for the vehicle control for the agonists, all dissolved in DMSO). **H** Five-minute pretreatment of the slices with the highly selective GPR55 antagonist CID16020046 (1 µM) also abolished the stimulatory actions of the GPR55 agonists AM251 (1 µM), LPI (100 nM), O-1602 (1 µM), and Δ^9^-THC (100 nM), i.e. at concentrations producing maximal stimulation when applied alone, but also suppressed JWH133 (1 µM)-induced glucose uptake (*n* = 9 rats per treatment). The leftmost bar shows the antagonist control condition (CBD 1 µM + DMSO, where DMSO stands for the vehicle control for the agonists). At 3 µM, CID unexpectedly increased glucose uptake (*n* = 9), despite showing no intrinsic effect at 1 µM. **I** BAPTA-AM loading (30 µM; 40 min) followed by washout prevented all agonists from stimulating glucose uptake (*n* = 6 each), implicating GPR55-G_q/11_-driven cytosolic Ca^2+^ elevation in glucose uptake stimulation. Data are mean ± SEM obtained from the indicated number of animals (*n* ≥ 5). *^+$^*P* < 0.05, **P < 0.01, ***P < 0.001, n.s., not significant. Missing error bars are within symbols

First, we started with the positive controls. We regularly use a relatively mild stimulus with high K⁺ (final K⁺ concentration: 20 mM) as a positive control to test slice responsiveness. High-K⁺ stimulation increased glucose uptake to 148.9 ± 6.3% of control (*n* = 28, *p* < 10^− 7^), with high fidelity. We have previously shown that CB_2_R-preferring ligands, JWH133 and GP1a increased both neuronal and astrocytic glucose uptake and metabolism in the mouse brain and elevated [^18^F]-fluorodeoxyglucose ([^18^F]FDG) PET signal, indicating potential therapeutic relevance for Alzheimer’s-related cerebral hypometabolism (Köfalvi et al. 2016). As an additional positive control, we now report that JWH133 (0.1–10 µM) significantly stimulated glucose uptake in the rat hippocampus, reaching up to 133.5 ± 8.5% of control (*n* = 6; *p* < 0.05; Fig. 1A).

The endogenous GPR55 agonist, LPI (1 nM–10 µM; *n* = 6–25), the phytocannabinoid Δ^9^-THC (1 nM–10 µM; *n* = 6–19), and the potent, synthetic GPR55 agonist, O-1602 (1 µM; *n* = 10) also produced statistically significant increases in glucose uptake (Fig. 1B, C, D). Nevertheless, none of these ligands produced classical sigmoidal concentration–response curves. This aligns with earlier pharmacological reports describing the atypical, non-sigmoidal nature of responses driven by GPR55 agonists (Anavi-Goffer et al. 2012).

At this point, one may wonder whether GPR55 agonists can only stimulate resting glucose uptake or also enhance glucose uptake in depolarized slices, on top of the KCl-induced uptake. To address this, we selected O-1602, as it is a selective GPR55 agonist, whereas Δ^9^-THC is not, and it is not subject to cellular uptake and degradation like LPI. We found that O-1602 (1 µM) significantly increased glucose uptake to 127.5 ± 16.4% of the KCl + DMSO control (*n* = 7; *p* < 0.05; Fig. 1E). These results indicate that GPR55 agonists can enhance glucose uptake in the brain both at rest and during neuronal activity.

The mixed CB_1_R-inverse agonist/GPR55 agonist AM251 (1 nM–10 µM; *n* = 6–41) also stimulated glucose uptake with the only difference being that this compound required a larger number of animals at 500 nM (*n* = 41) to reach conclusive results, because 37–38% of the animals were considered non-responders (AM251 effect ≤ 5% increase). While AM251 displays high affinity for CB_1_R (IC₅₀ ≈ 8 nM), its affinity for GPR55 lies in the high-nanomolar to low-micromolar range (Ryberg et al. 2007; Kargl et al. 2013), as also reflected in our study. Consistent with this profile, AM251 significantly stimulated glucose uptake only from 500 nM upward, reaching an *E*_max_ of 125.8 ± 9.0% (*n* = 11; *p* < 0.01; Fig. 1F).

At this point, one may claim that AM251’s stimulatory effect might be related to disinhibition of mitochondrial CB_1_Rs (mtCB_1_Rs), which are known to affect glucose metabolism in astrocytes (Bénard et al. 2012; Duarte et al. 2012; Harkany and Horvath 2017), with consequences on cell survival and social behavior (Jimenez-Blasco et al. 2020). To explore this possibility, we tested four additional CB_1_R antagonists at concentrations of 100 nM, 500 nM, and 1 µM (*n* = 6–12): (i) the AM251 analogues CP945598 (highly selective and potent CB_1_R antagonists/inverse agonists, also known as Otenabant; Kim et al. 2008) and (ii) PF514273 (another highly selective and potent CB_1_R antagonists/inverse agonists; Dow et al. 2009), (iii) the structurally dissimilar and less CB_1_R-selective antagonist/inverse agonist LY320135, and (iv) the highly CB_1_R-selective neutral antagonist O-2050.

  In these experiments, none of these CB_1_R antagonists significantly modulated glucose uptake (fig. not shown). Notably, another CB_1_R antagonist (not tested here), AM281, differs from AM251 by the substitution of a carbon atom with an oxygen atom in its pyridine moiety, resulting in a 1,4-oxazine-like heteroaromatic ring. Despite this subtle modification, AM281 is inactive at the human GPR55 receptor (Ryberg et al. 2007; Kapur et al. 2009). Therefore, it is not surprising that more substantial structural modifications to AM251 – as seen in CP945598 and PF514273 – abolished their effect on glucose turnover in our assay. These data altogether suggest a lack of appreciable endocannabinoid tone at mtCB_1_Rs in the resting hippocampal slices and that the effect of AM251 was unlikely to be CB_1_R-dependent in our assay.

CBD (10 nM–10 µM; *n* = 6–8), the natural inhibitor of cannabinoid receptors including the GPR55, also had no effect on its own. Figure 1G illustrates CBD alone at 1 µM (leftmost bar). However, pretreatment of slices with 1 µM CBD for 5 min fully prevented the stimulatory action of LPI (100 nM; *n* = 6), Δ^9^-THC (100 nM; *n* = 6), and AM251 (1 µM; *n* = 6) (Fig. 1G). Although the similar stimulatory effect on glucose uptake of two GPR55-selective agonists (LPI and O-1602) and two non-selective GPR55 agonists (Δ^9^-THC and AM251) strongly suggest GPR55 involvement, a definitive proof would be the preventive action of a GPR55-selective antagonist, such as CID16020046 (CID; Kargl et al. 2013).

While CID is a highly selective GPR55 antagonist, it fully antagonizes AM251- and LPI-induced signalling only in the low-micromolar range (Kargl et al. 2013). In our hands, CID produced no appreciable effect on glucose uptake at 1 µM (*n* = 9; Fig. 1H, leftmost bar). However, unexpectedly, it strongly increased glucose uptake at 3 µM by 63.4 ± 19.9% (*n* = 9; *p* < 0.05; Fig. 1H). Whether this reflects unanticipated agonism at GPR55, circuitry-mediated modulation, or an off-target mechanism remains to be determined. Nevertheless, CID (1 µM), applied 5 min before ligand exposure, fully prevented the maximal stimulatory action of AM251 (1 µM), LPI (100 nM), O-1602 (1 µM), and Δ⁹-THC (100 nM) – that is, at concentrations where each agonist individually reached its maximal effect (Fig. 1H). An additional unexpected observation was the blockade of the response to the CB_2_R-preferring agonist JWH133 (Fig. 1H).

In theory, intracellular Ca^2+^ elevation is sufficient to stimulate glycolytic enzyme activity and lactate release with a consequent concomitant increase in glucose uptake, in astrocytes (Horvat et al. 2021). GPR55 is known to signal via G_q_, leading to PLCβ activation, IP_3_ formation, and subsequent Ca^2+^ release from intracellular stores (Lauckner et al. 2008). Hence, this signaling cascade could represent a plausible explanation for the stimulation of glucose uptake upon GPR55 activation in the slices. To test this hypothesis, hippocampal slices were preincubated with the membrane-permeable Ca^2+^ chelator, BAPTA-AM (30 µM) for 40 min, followed by 20 min washout and subsequent exposure to cannabinoid agonists.

BAPTA-pretreatment did not alter basal glucose uptake rates (*n* = 6; Fig. 1I, leftmost bar), but it fully abolished the stimulatory effects of AM251 (1 µM), LPI (100 nM), O-1602 (1 µM), Δ⁹-THC (100 nM), and JWH133 (1 µM) (*n* = 6 for each ligand). After BAPTA-AM pretreatment, most ligands exhibited a modest, non-significant reduction of basal glucose uptake rather than stimulation.

### *Gpr55* gene expression in astrocytes in culture

**Fig. 2:**
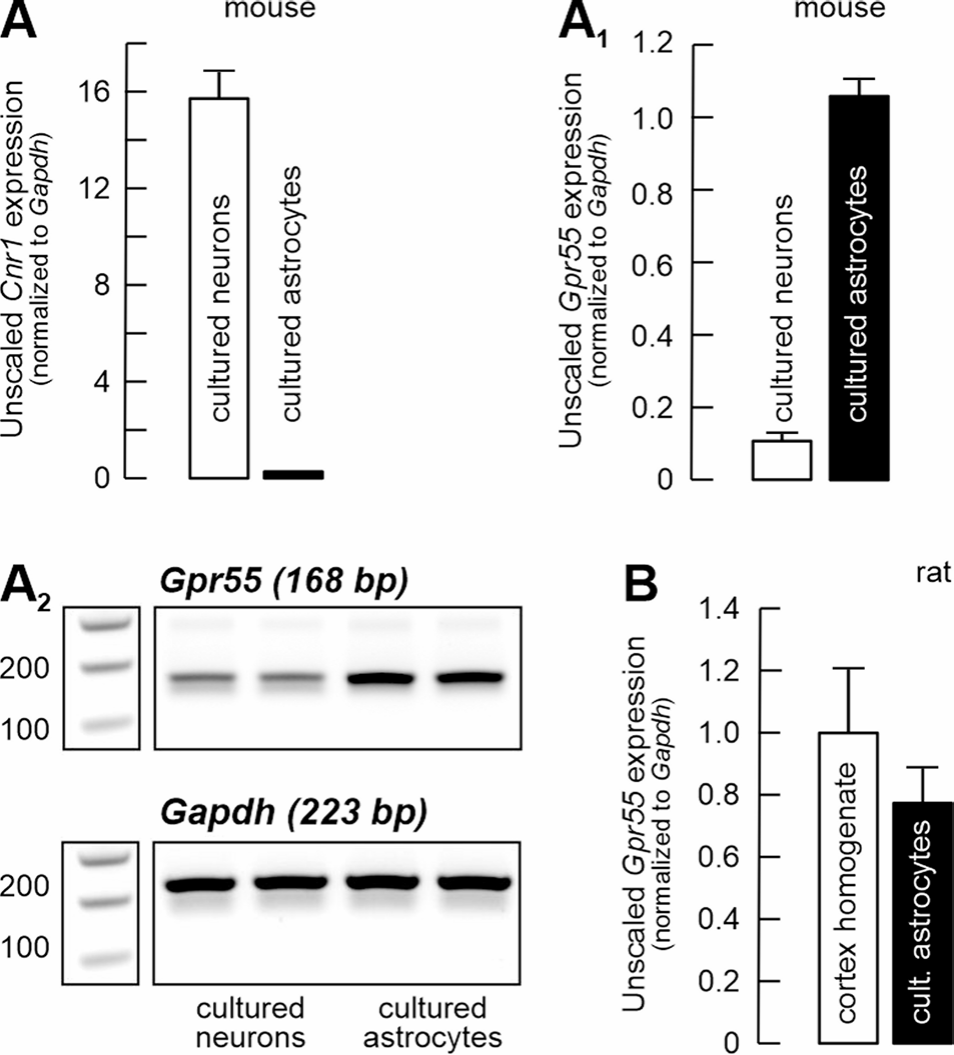
Gpr55 mRNA is predominantly astrocytic. Bar graphs summarizing Cnr1 (**A**) and Gpr55 (**A**_**1**_) mRNA levels, normalized to the housekeeper Gapdh, in mouse cortical neuronal and astrocytic cultures. **A**_**2**_) Representative gels illustrating relative transcript abundance. Importantly, expression differences were quantified from cycle threshold values during the exponential phase of amplification, not from the final gel bands after 40 cycles. For further details, see Supplementary Fig. 1. **B**) Gpr55 mRNA levels, normalized to Gapdh, are comparable between cortical homogenates of 2 month-old rats and rat astrocytic cultures, indicating that in rats as well, Gpr55 expression is predominantly astrocytic

The systematic comparative analysis of the exact cellular expression and distribution of GPR55 has not been reported yet, but it is quite likely that GPR55 expression is widespread in the mammalian brain, including humans (Solymosi and Köfalvi, 2017; Martínez-Pinilla et al. 2020; Menéndez-Pérez et al. 2024; Rodrigues et al. 2024). Figure 2 summarizes our results on *Gpr55* mRNA expression levels in mouse primary neurons and astrocytes in culture (A–A_2_), in rat cortical homogenates, as well as in rat cortical astrocytes (B). *Cnr1* and *Gpr55* mRNA expressions appeared to be complementary in cultured cortical neurons and astrocytes, with *Gpr55* being dominant in astrocytes (Fig. 2A1). Meanwhile, in adult rat cortical homogenates, the levels of *Gpr55* mRNA were comparable to those from cultured astrocytes (Fig. 2B).

### High resolution respirometry reveals no modulation of mitochondrial activity by LPI

Astrocytes predominantly engage in aerobic glycolysis, releasing lactate that is shuttled to neurons. Neurons convert lactate to pyruvate in the cytosol, and pyruvate is then oxidized in mitochondria to fuel oxidative phosphorylation. Although neurons can take up glucose, a significant fraction is diverted to the pentose phosphate pathway rather than being oxidized directly, emphasizing a metabolic division of labor between astrocytes and neurons (Wyss et al. 2011; Pellerin and Magistretti 2012; Lerchundi et al. 2015; Lundgaard et al. 2015; Barros et al. 2023). Therefore, the predominant astrocytic expression of GPR55 hints that GPR55 activation could stimulate astrocytic lactate production rather than glucose complete oxidation. To test this hypothesis, we first measured tissue oxidative metabolism using oxygen consumption as a proxy in whole and intact hippocampal slices.

**Fig. 3:**
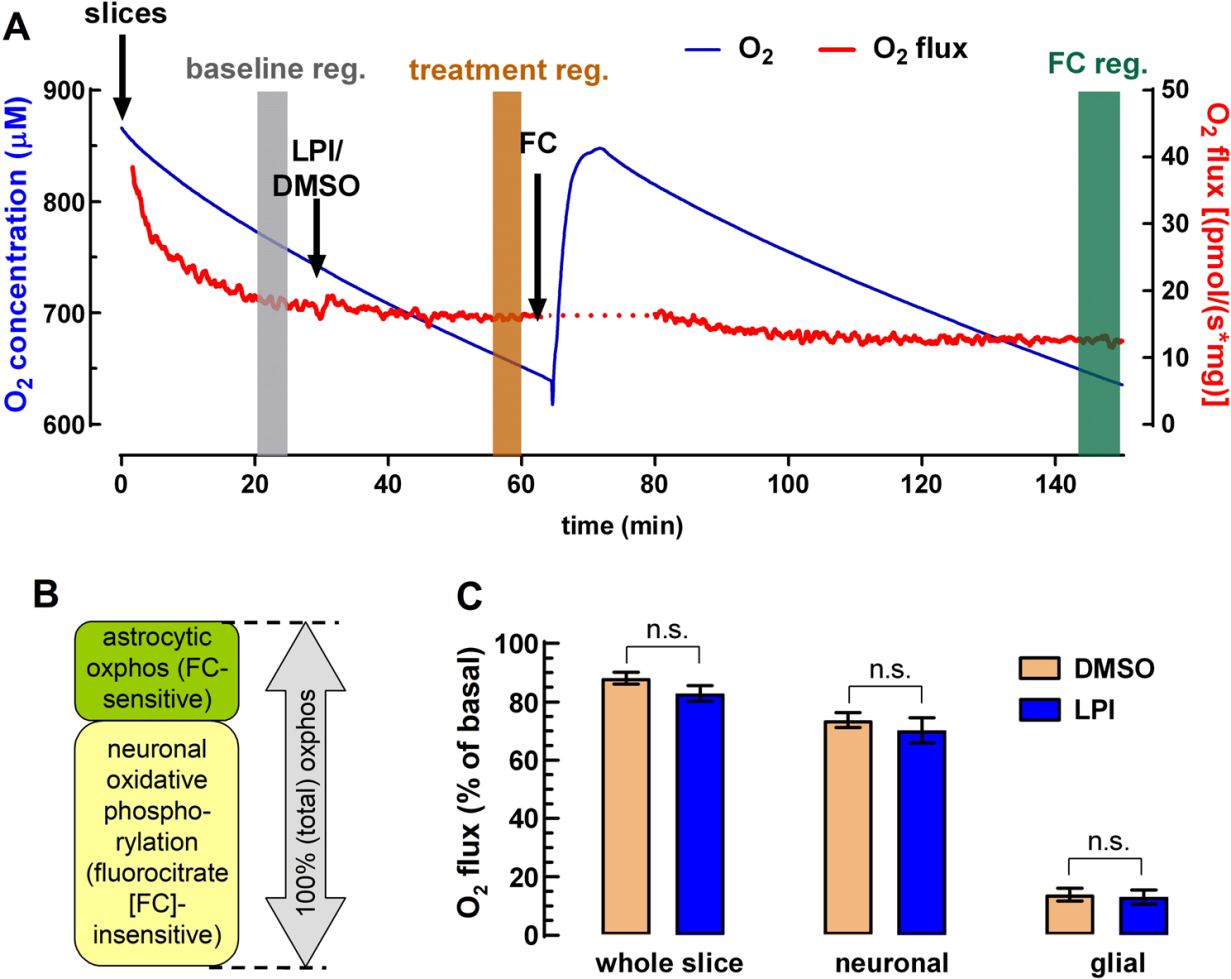
LPI does not significantly affect oxidative metabolism in neuronal and glial compartments. **A** Representative trace of oxygen flux (red) plotted against oxygen concentration (blue) over time. Arrows indicate the addition of LPI (100 nM), its vehicle (DMSO), and fluorocitrate (FC). Colored bars mark the segments selected for analysis (i.e. baseline, treatment and fluorocitrate registration). **B** Schematic representation illustrating that total oxidative phosphorylation (oxphos) is composed primarily of a neuronal (FC-insensitive, ~ 84%) and a smaller glial (FC-sensitive, ~ 16%) component. **C** Comparison of oxphos between LPI-treated slices (blue bars) obtained from *n* = 10 rats and vehicle controls (beige bars) (*n* = 11), shown for total tissue, neuronal, and astrocytic compartments

Basal respiration in hippocampal tissue was determined after leaving the slices undisturbed in the holding chamber for ~ 30 min (Fig. 3A). The O₂ flux obtained at this point was used for normalization. Next, the effect of LPI (100 nM) on tissue oxygen consumption was compared to DMSO control. As already expected, LPI treatment did not affect O₂ flux in the slices (Fig. 3A and C). Subsequently, we determined the relative contribution of neuronal (84.2 ± 2.4% of total) and non-neuronal (glial; 15.8 ± 2.4% of total) fractions to the total oxidative metabolism in the slice with the addition of sodium fluorocitrate (100 µM) (Fig. 3B and C). Fluorocitrate is preferentially taken up by glia and inhibits the glial enzyme aconitase in the Krebs–Szent-Györgyi cycle (Fonnum et al. 1997). Altogether, these results suggest that LPI does not alter either neuronal or glial oxidative metabolism in the hippocampus, at its GPR55-selective concentrations tested.

### GPR55 activation stimulates glucose uptake and metabolism in cultured astrocytes

**Fig. 4:**
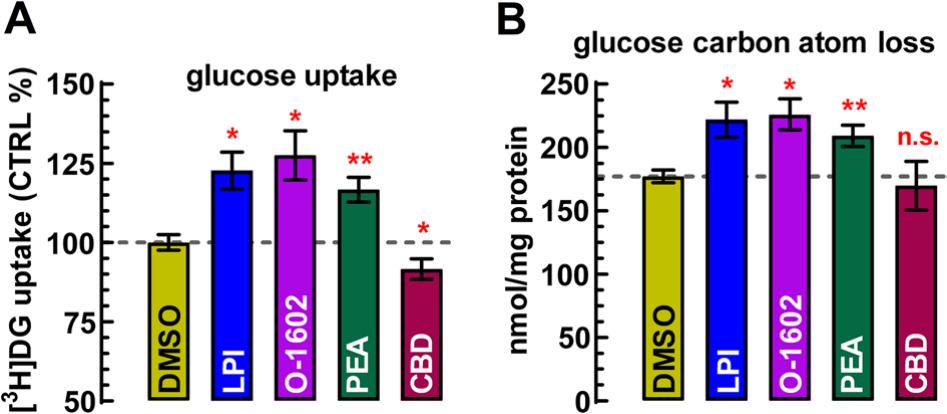
Glucose turnover is significantly stimulated upon GPR55 activation in astrocytes. **A** The GPR55 agonists LPI (100 nM), O-1602 (100 nM), and the endogenous ligand PEA (100 nM) all significantly stimulated [^3^H]DG uptake in primary astrocyte cultures (*n* = 6 independent cultures, each in quadruplicate). CBD (1 µM) slightly reduced glucose uptake, potentially due to inverse agonism or blockade of endogenous GPR55 activity

Glucose uptake amounted to 36.4 ± 3.1 nmol/mg protein under control conditions in cell cultures during a 30-minute incubation period. Since resting slices exhibited almost twice as high glucose uptake per mg protein (see above), it is evident that even under basal conditions, neuronal glucose uptake prevailed over that in astrocytes. Furthermore, glucose-derived carbon atom loss amounted to 177.0 ± 4.9 nmol/mg protein (Fig. 4B), suggesting that more than 80% of the carbon atoms derived from newly taken up glucose was released back into the assay medium, presumably as either CO₂ or lactate, or both. Figure 4A illustrates that LPI (100 nM) and PEA (100 nM), as well as O-1602 (100 nM), uniformly stimulated glucose uptake and, proportionally, dissipative glucose metabolism by approximately 20–25% above control levels (*p* < 0.05), while the GPR55 antagonist CBD (1 µM) exerted a modest inhibitory effect on glucose uptake.

### [^1^H]-NMR spectroscopy unveils GPR55-induced Glycolysis in astrocytes

**Fig. 5:**
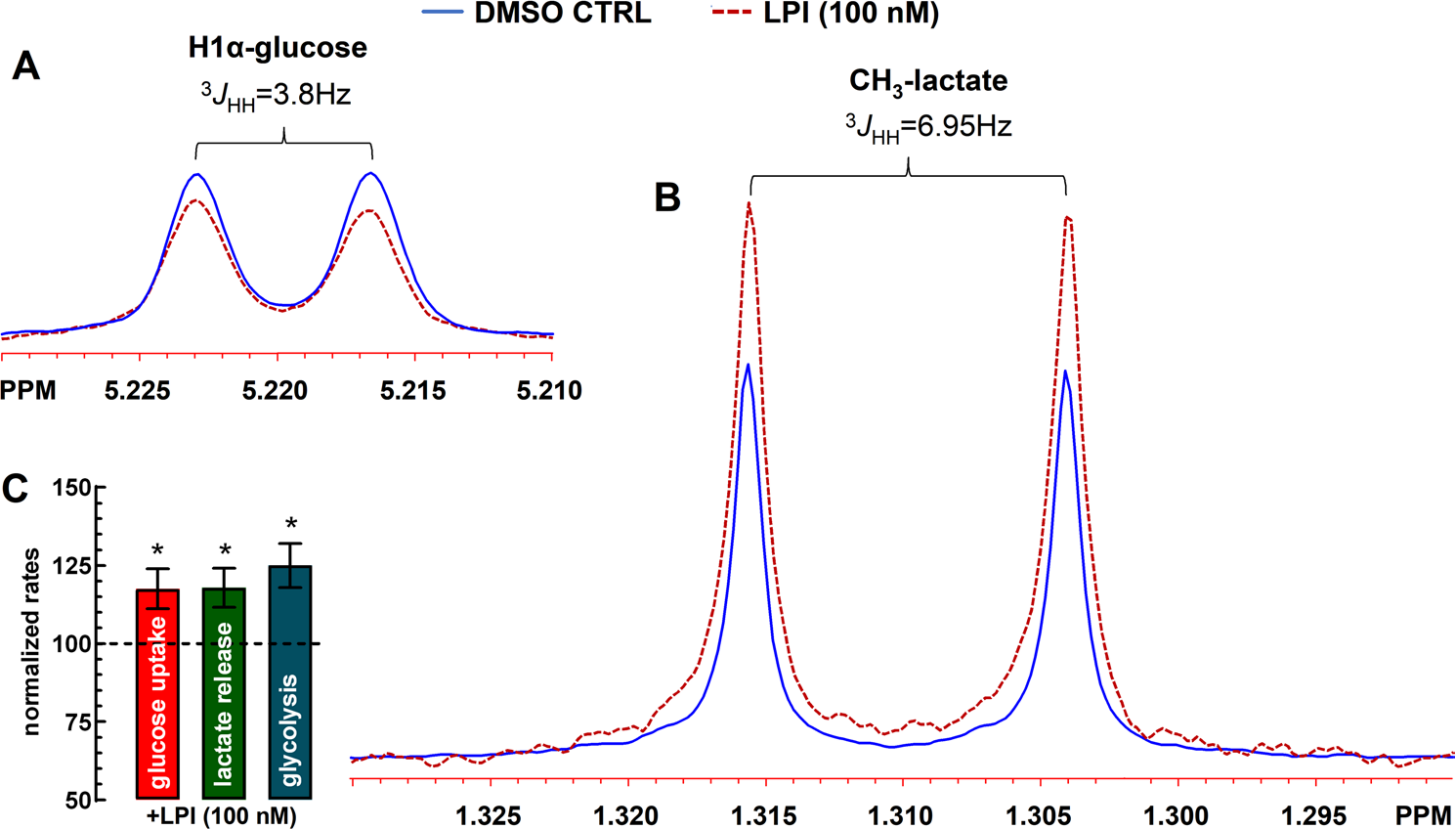
The endogenous GPR55 agonist, LPI (100 nM) stimulates glucose uptake, glycolysis and lactate release in rat cortical astrocytes. **A**,** B** The X-axis labeled in ppm (parts per million) represents chemical shift, which illustrates the electronic environment of the proton (¹H). Multiplets (splitting patterns) occur due to scalar coupling between nearby hydrogens. The coupling constant, labeled ³*J*_HH_, is measured in Hz and reflects the interaction between two protons three bonds apart. H1α-glucose resonance or α-anomer of glucose refers to a specific cyclic form of glucose where the hydroxyl group at the anomeric carbon (carbon 1) is in the axial position (pointing down). In this configuration, the anomeric hydroxyl group is on the same side of the ring as the CH₂OH group on carbon 5. The splitting constant ³*J*_HH_ = 3.8 Hz is a fingerprint for an axial-equatorial coupling in a pyranose ring (like H1α-glucose). The position (chemical shift in ppm) is usually around 5.2–5.5 ppm for H1α in aqueous solution. This signature doublet and coupling constant are diagnostic signals of D-glucose. Lactate was identified by the characteristic CH_3_ doublet at δ ~ 1.31 ppm (³*J*_HH_ = 6.95 Hz). The coupling constant of 6.95 Hz is typical for a 3-bond coupling in such a structure, while the methyl group of lactate, which is coupled to the methine proton (-CH(OH)-CH₃), shows up as a doublet for the resonance of the methyl group (-CH₃ coupled to one proton), usually near 1.3 ppm. Absolute D-glucose consumption and lactate production were determined by comparison of the intensity of the resonances due to glucose (H1α) and lactate (-CH_3_) with the resonance of the used internal standard (fumarate 2 mM), taking into consideration the number of protons contributing to each resonance. **C** Bar graph summarizing the calculated glucose uptake, lactate release and glycolytic rates for *n* = 5 independent astrocytic cultures in triplicates, *p < 0.05

Figure 5 shows the reduction in glucose and increase in lactate levels in the incubation medium after 30-min, as compared to the DMSO control. LPI (100 nM) significantly reduced extracellular glucose levels – interpreted as increased glucose uptake (Fig. 5C) – and enhanced lactate production. The calculated increase in the aerobic glycolytic rate was 125.0 ± 7.0% of control (Fig. 5C). For measurement details, see the figure legend. Altogether, our findings suggest that GPR55 activation on cultured astrocytes stimulated glucose uptake, glycolysis, and lactate output.

## Discussion

### The importance of studying brain glucose metabolism

Glucose is the brain’s principal fuel, accounting for roughly 20–25% of whole-body glucose use. In the resting state, neurons consume most of this glucose through oxidative phosphorylation, with the majority of the resulting ATP devoted to ion pumping and glutamatergic transmission (Beltrán et al. 2012; Magistretti and Allaman 2013). Astrocytes respond to small rises in extracellular K⁺ and glutamate by reducing oxidative metabolism, activating glycogenolysis, and releasing lactate, which neurons convert to pyruvate. This aerobic glycolysis (the astrocyte–neuron lactate shuttle or ANLS in short) supports neuronal activity while sparing oxygen for neuronal oxidative phosphorylation (Dienel 2019; Barros et al. 2023; Dias et al. 2023; Pellerin and Magistretti 2012) and is required for synaptic plasticity and memory (Suzuki et al. 2011). However, alternative views propose that active neurons can also take up their own glucose and engage in glycolysis (Lundgaard et al. 2015; Díaz-García et al. 2017).

Alterations in [^18^F]FDG uptake are early markers of brain dysfunction (Zhang et al. 2021), and virtually all major neurological and psychiatric disorders display characteristic FDG-PET signatures (Sukumar et al. 2020; Ko and Strafella 2022; Triumbari et al. 2024; Wang et al. 2025; Zarovniaeva et al. 2025). We advocate for a deeper understanding of the physiological signals (such as endocannabinoids) that regulate cerebral energy metabolism and the mechanisms through which they act, so that maladaptive signaling can be targeted to correct metabolic dysfunction and potentially mitigate the progression of brain disorders (de Ceballos and Köfalvi 2017).

### Astrocytic GPR55 receptors fuel glycolysis

The present study characterizes a previously unrecognized function of the third cannabinoid receptor, the GPR55, in the central nervous system – the regulation of cellular energetics. We demonstrated that multiple GPR55 agonists significantly increased glucose uptake and its conversion to lactate in acute brain slices and primary astrocyte cultures. The magnitude of this response was comparable to that elicited by positive controls. GPR55-mediated stimulation of glucose uptake was statistically significant across brain regions and was conserved between species, remaining detectable at agonist concentrations as low as 10 nM.

The GPR55-selective antagonist/inverse agonist CID abolished the glucose-uptake responses induced by AM251, LPI, O-1602, and Δ^9^-THC, strongly supporting a GPR55-dependent mechanism. Given that qPCR analyses revealed predominantly astrocytic GPR55 expression – nearly complementary to neuronal CB_1_R – we validated our key findings in primary astrocyte cultures. GPR55 activation also stimulated glycolysis and lactate release in astrocytes – as shown with proton nuclear magnetic resonance ([^1^H]-NMR) spectroscopy, rather than the oxidative metabolism of glucose in neurons or astrocytes – as gauged with high-resolution respirometry.

In parallel, cytosolic Ca^2+^ chelation fully prevented GPR55-agonist-induced metabolic stimulation. GPR55 is known to signal via Gα_q/11_, leading to PLCβ activation, IP_3_ formation, and subsequent Ca^2+^ release from intracellular stores, mainly endoplasmic reticulum (Lauckner et al. 2008). A recent study showed that a rise in cytosolic Ca²⁺ engages astrocytic glycolysis (Horvat et al. 2021). Thus, it is reasonable to conclude that GPR55 activation in astrocytes enhances glycolysis and lactate release via increase in cytosolic Ca²⁺ levels, which in turn is sufficient to stimulate secondary glucose uptake.

Given that astrocyte-derived lactate is required for LTP induction and memory consolidation (Suzuki et al. 2011; Fernández-Moncada et al. 2024), our finding that GPR55 activation rapidly stimulates lactate release suggests that GPR55 may function as an additional metabolic gate for synaptic plasticity. In this view, GPR55-driven glycolysis could complement endocannabinoid-induced metabolic signaling, ensuring adequate lactate availability during periods of high network demand. Such a mechanism would position GPR55 as an accessory modulatory pathway that supports the energetic requirements of LTP and memory formation.

### CB_1_R activation has been shown to modulate glucose metabolism in the brain

CB_1_R is by far the most abundant cannabinoid receptor in the brain (Ligresti et al. 2016; Araque et al. 2017; Piazza et al. 2017), and therefore, if CB_2_R and GPR55 participate in regulating brain energy metabolism, it is reasonable to expect that CB_1_R does as well. Indeed, earlier observations establish that CB_1_Rs located at the outer mitochondrial membrane (mtCB_1_Rs) suppress the activity of mitochondrial complex I, thereby decreasing respiratory flux and ATP production (Bénard et al. 2012; Duarte et al. 2012; Harkany and Horvath 2017). This metabolic brake feeds back onto synaptic physiology by weakening the energetic support for neurotransmission, ultimately shaping synaptic plasticity, learning, and memory.

In astrocytes, mtCB_1_R signaling similarly downregulates complex I activity but with broader metabolic consequences: it diminishes glycolysis, lactate production, and lactate shuttling to neurons, thereby altering neuronal redox balance and impairing complex behaviors such as social interaction (Jimenez-Blasco et al. 2020). In our hands, direct CB_1_R activation or inhibition in resting hippocampal slices did not affect [^3^H]DG uptake (Lemos et al. 2012; present study). This may reflect the limited temporal resolution of our 30-min uptake protocol, which is unlikely to detect rapid (≤ 5 min) metabolic transients, or the fact that resting slices exhibit minimal energy demand to be suppressed by CB_1_R activation (or both). Indeed, only when we increased network activity with 4-aminopyridine did the CB_1_R/CB_2_R agonist WIN55212-2 reduce mitochondrial intermediary metabolism, as revealed by [^13^C]-NMR isotopomer analysis in a manner prevented by CB_1_R antagonism (Duarte et al. 2012). Notably, this isotopomer approach has a very low temporal resolution, requiring a 3-hour incubation with [^13^C]-glucose and [^13^C]-acetate to reach metabolic steady state.

In contrast, a recent study employing a high-temporal resolution lactate sensor assay besides several cutting-edge molecular, genetic, physiological and behavioral approaches revealed that astroglial CB_1_Rs have a biphasic and compartment-specific control of lactate, rather than simply “increasing” or “decreasing” it (Fernández-Moncada et al. 2024). Short-term (~ 5 min) activation of non-mitochondrial CB_1_Rs on astrocytes rapidly boosts glycolysis and lactate release, which then acts on the lactate receptor GPR81 (or HCAR1) to bias glycolysis toward the phosphorylated pathway, increase L- and D-serine production, enhance NMDAR co-agonist occupancy, and support novel object recognition memory. In contrast, prolonged activation of mtCB_1_R depresses complex I as discussed above, hence reducing lactate production, inducing neuronal energetic stress, and impairing behavior.

CB_1_R-mediated G_q/11_ signaling in astrocytes is well known to elevate cytosolic Ca^2+^ and trigger glutamate release (Ligresti et al. 2016; Araque et al. 2017). Beyond gliotransmission, rise in [Ca^2+^]_i_ (elicited by either cannabinoid receptor) is sufficient to stimulate glycolysis in astrocytes, as discussed above (Horvat et al. 2021). Together with the short-term lactate-stimulating effects of astroglial CB_1_Rs described by Fernández-Moncada et al. (2024) these findings support the existence of a straightforward, activity-dependent metabolic mechanism whereby endocannabinoid release at the quadripartite synapse transiently enhances lactate output to sustain the energetic demands of cognition. The engagement of additional cannabinoid receptors, such as CB_2_R and GPR55, likely broadens the signaling repertoire and provides redundancy through receptor-selective endocannabinoids, thereby increasing the robustness of astrocyte metabolic responses.

### Considerations regarding CB_2_R involvement in cerebral glucose metabolism

We previously demonstrated in mouse brain slices, mouse cortical astrocyte cultures, and mouse brain microPET assays that two structurally dissimilar CB_2_R-preferring agonists (GP1a and JWH133) stimulate glucose uptake in a manner sensitive to the CB_2_R antagonist AM630 (Köfalvi et al. 2016). Curiously, in the present study, the stimulatory effect of JWH133 was sensitive to both the GPR55 antagonist CID and cytosolic Ca^2+^ chelation in rat hippocampal slices. If astrocytes are indeed endowed with CB_2_Rs, cross-talk between CB_2_R and GPR55 could occur via heteromeric receptor complexes. Indeed, GPR55 has been shown to form heteromeric assemblies with CB_2_R in the brain (Balenga et al. 2014; Martínez-Pinilla et al. 2020; Menéndez-Pérez et al. 2024), including in glioblastoma cells (*i.e.*, transformed astrocytes) (Moreno et al. 2014), resulting in substantial functional interactions.

GPCR heteromers often display integrated pharmacology, in which ligand binding to either protomer can modulate the signaling of the entire complex. Agonists may activate the heteromer through direct or cross-activation, while antagonists of one receptor can allosterically suppress signaling of the partner receptor. If JWH133 engages a CB_2_R–GPR55 heteromer, this could explain why Δ^9^-THC fails to stimulate glucose uptake via CB_2_R pathways when GPR55 is pharmacologically blocked: inhibition of the GPR55 protomer would be expected to silence CB_2_R-dependent signaling within the heteromer, analogous to the cross-inhibitory effect of adenosine A_2A_R antagonists on CB_1_R signaling in the A_2A_R–CB_1_R heterotetramer (Köfalvi et al. 2020).

### Word of caution regarding astrocytic CB_2_Rs

Several recent studies urge caution when attributing metabolic effects of cannabinoid ligands to CB_2_Rs in the brain, particularly in astrocytes. Savonenko et al. (2015) reported that in healthy mouse cortex CB_2_R immunoreactivity is predominantly neuronal, whereas astrocytic CB_2_R labeling is negligible and microglial CB_2_R-positive ROIs are rare. In contrast, in mouse models of β-amyloidosis, CB_2_R signal becomes markedly upregulated in microglia and, to a lesser extent, in astrocytes, consistent with a disease- and activation-dependent expression pattern. These observations argue against substantial CB_2_R expression in non-reactive astrocytes under physiological conditions and highlight the strong dependence of CB_2_R detectability on brain state.

In agreement with this view, Fink et al. (2025) failed to detect *Cnr2* transcripts in cultured neonatal astrocytes, while clearly identifying *Cnr1*. In their study, the CB_1_R-selective agonist ACEA triggered a rapid and transient rise in intracellular Ca^2+^, glucose, and lactate, peaking within approximately 5 min, thus fully recapitulating and confirming the findings of Fernández-Moncada et al. (2024). By contrast, two CB_2_R-preferring ligands, GP1a and AM1241, elicited slower-onset and more sustained increases in intracellular glucose and lactate, developing over tens of minutes. In the absence of detectable *Cnr2* expression, Fink et al. concluded that GP1a and AM1241 likely acted through receptors other than CB_2_R.

This interpretation is further supported by recent large-scale pharmacological profiling. Soethoudt et al. (2017) demonstrated that GP1a does not behave as an agonist but rather as an inverse agonist (*i.e.*, antagonist) at CB_2_R, while showing no agonist activity at CB_1_R. The considerable structural similarity between GP1a and AM251 also raises the speculative possibility that GP1a could potentially engage the GPR55; however, this hypothesis remains untested and should be interpreted with caution. 

Moreover, several other CB_2_R-preferring ligands, including JWH133 and AM1241, display limited selectivity and pronounced context dependence in rodent systems, and may also affect enzymes and transporters involved in endocannabinoid turnover (Soethoudt et al. 2017). Indeed, Anavi-Goffer et al. (2012) reported that JWH133 is also active at GPR55; however, it behaved as an inverse agonist in transfected HEK293 cells lacking CB_2_R. Nevertheless, we emphasize that JWH133 was included primarily as a historical positive control, based on our previous demonstration that this ligand enhances brain glucose uptake in vivo and ex vivo in the mouse brain (Köfalvi et al. 2016), rather that testing the functional presence of the CB_2_R.

### Therapeutic significance of GPR55-mediated astrocytic lactate release

The implications of our findings are multifaceted. Central GPR55 has been proposed as a major therapeutic target of the CBD formulation Epidiolex (Sekar and Pack 2019) in certain treatment-resistant epilepsies. Epidiolex reduces seizure frequency in large part because CBD restores the excitatory/inhibitory balance through the blockade of presynaptic GPR55 and the modulation of intracellular Ca^2+^ levels (Sylantyev et al. 2013; Kaplan et al. 2017; Gray and Whalley 2020; Rosenberg et al. 2023; Borowicz-Reutt et al. 2024). Epilepsy is relevant to our study because impaired neuron–astrocyte glucose metabolic crosstalk is a widely recognized epileptogenic factor (Santucci et al. 2024; Dhureja et al. 2025). Both diabetes and glucose transporter deficiency can contribute to epileptogenesis (Santucci et al. 2024; Saini and Panchal 2025). Excessive lactate accumulation can also be pro-epileptogenic via increased protein lactylation (Kuang et al. 2025). On the other hand, lactate, through activation of its receptor GPR81 (also designated as HCAR1), is also able to reduce neuronal excitability and astrocytic energy production (de Castro Abrantes et al. 2019; Chen et al. 2025).

It is possible that astrocytic GPR55 receptors become active during seizures to support the heightened neuronal energy demand. There is indirect evidence suggesting that LPI is released into the extracellular space upon neuronal activity, particularly via increased Ca^2+^ signaling and the activation of cytosolic phospholipase A_2_ (Alhouayek et al. 2018). However, to our knowledge, no study has directly demonstrated the synaptic release of LPI in response to stimulation, as has been shown for glutamate or 2-AG.

One could argue that the contribution of LPI to lactate release was relatively modest (20–25%) in our study, albeit statistically significant. Nevertheless, seizures increase GPR55 expression, allowing this receptor to exert greater control over circuit excitability in the hippocampus (Gray and Whalley 2020; Rosenberg et al. 2023; Borowicz-Reutt et al. 2024). Moreover, hippocampal sclerosis and astrogliosis may further amplify GPR55-mediated lactate release in chronic epilepsy, due to the increase in astrocyte-to-neuron ratio, contributing to protein lactylation with consequences on neuronal excitability. Additional studies are needed to determine whether LPI or other endogenous GPR55 ligands are released in an activity-dependent fashion during seizures and whether GPR55-mediated lactate release becomes more prominent in advanced epileptic stages.

Another major brain pathology characterized by profound metabolic reprogramming and increased lactate production is glioblastoma , *i.e.* (Calvert et al. 2017; Stanke et al. 2021). Glioblastoma is the most aggressive and treatment-resistant brain tumor, with a median survival of just 15 months despite standard therapy (Chen et al. 2017). It exhibits elevated glucose uptake, which facilitates its detection with [^18^F]FDG PET imaging (Wang et al. 2025), and vastly increased lactate production. Lactate is, in fact, the most powerful oncometabolite of glioblastoma, produced under normoxic conditions via a form of metabolic reprogramming known as the Warburg effect (Liberti et al. 2016). Together with tissue acidity, lactate supports stemness, migration, survival, and energy production in glioma cells (San-Millán and Brooks 2017). Consequently, the Warburg effect is also considered the Achilles heel of glioblastoma.

Once again, we refer to GPR55 blockade as a putative therapeutic strategy to reduce lactate release and glioblastoma survival. GPR55 expression correlates strongly with cancer aggressiveness, activating Rho/ROCK, MAPK, ERK, NF-κB, and PI_3_K/Akt signaling pathways, all of which are hyperactive in glioblastoma (Ford et al. 2010; Andradas et al. 2011; Bernier et al. 2017; Calvillo-Robledo et al. 2022). Moreover, LPI secretion is mediated by the ABCC1 (MRP-1) transporter (Alhouayek et al. 2018), a multidrug resistance protein frequently upregulated in aggressive tumors. This forms an autocrine GPR55–ABCC1–LPI loop that may drive malignancy and chemoresistance (Piñeiro et al. 2011; Ross 2011). GPR55 expression is elevated in astrocytoma and glioma cell lines, as well as in patient-derived tissue samples (Andradas et al. 2011), and GPR55 agonists such as Δ^9^-THC and LPI reduce the number of Ki67-positive nuclei in glioblastoma cells (Kolbe et al. 2021), possibly reflecting a shift toward therapy-resistant glioma stem cells.

While high Ki67 expression typically reflects a highly proliferative and aggressive tumor, low Ki67 expression does not necessarily indicate a better prognosis, especially in glioblastoma. In this context, cellular quiescence and stemness – potentially induced by extracellular lactate accumulation – contribute to resistance against chemotherapy and radiotherapy. Altogether, our hypothesis is that upregulated GPR55 signaling sustains tumor progression by promoting metabolic plasticity, stemness, and drug resistance. Even if GPR55 activation produces only a modest (≤ 25%) increase in glucose uptake and lactate release in slices and cultured astrocytes, the strong upregulation of GPR55 in glioblastoma may significantly contribute to a shift in metabolic programming from the Crabtree effect to the Warburg effect. This could explain why selective GPR55 blockade reduces chemoresistance in cancer cells (Singh et al. 2016), and more specifically, why cannabidiol exhibits antiglioma activity (Vaccani et al. 2005; Lah et al. 2022).

### Limitations of this study

It is important to exert caution when interpreting expression levels. First, our cultured cells are derived from embryonic and newborn pups, and their mRNA profiles may not accurately reflect adult receptor expression. Moreover, high GPR55 and low CB_1_R mRNA levels do not necessarily mirror their respective protein levels. For example, we previously observed that low CB_1_R mRNA levels were accompanied by increased protein levels in the hippocampus of diabetic rats (Duarte et al. 2007). This can occur because efficiently translated mRNAs are often degraded more rapidly, resulting in low steady-state mRNA levels. Translation exposes mRNAs to deadenylation and decapping enzymes, especially in the presence of specific RNA-binding proteins or microRNAs. In some cases, the protein itself can influence the stability of its own mRNA.

It is also important to keep in mind that in acute brain slices or in whole-brain live imaging e.g. with [^18^F]FDG-PET, cell-type attribution to cannabinoid effects becomes inherently more complex. Although our data suggest that CB_1_R mRNA is predominantly neuronal while GPR55 is mostly expressed in astrocytes, there is ample literature evidence supporting the presence of astrocytic CB_1_Rs and neuronal GPR55 receptors (see e.g. Araque et al. 2017; Solymosi and Köfalvi, 2017; Fernández-Moncada et al. 2024; Fink et al. 2025).

Recent evidence indicates that microglia, which can express significant CB_2_R levels, can contribute substantially to cerebral glucose uptake (Xiang et al. 2021; Kunte et al. 2025), although in healthy tissue their CB_2_R-expression and overall contribution to glucose uptake are likely constrained. Oligodendrocytes are also well known to engage in dynamic metabolic coupling with axons, and, several lines of evidence indicate that the CB_1_R, the CB_2_R and the GPR55 are present along the oligodendrocyte lineage (Ilyasov et al. 2018; Moreno-Luna et al. 2021). Still, quantitative estimates of their contribution to total cerebral glucose uptake remain scarce (Narine and Colognato 2022).

Altogether, a more holistic view of the endocannabinoid system is required to advance this field. As evidence accumulates for the involvement and subcellular localization of cannabinoid receptors in cerebral energy metabolism, discrepancies among studies have become increasingly apparent. These differences likely reflect the limited selectivity of many ligands, as well as variations in experimental models, concentration ranges, and exposure times (Soethoudt et al. 2017).

In addition, heteromerization among cannabinoid receptors must be considered in multipharmacological approaches such as the present study, since CB_1_R–GPR55, CB_1_R–CB_2_R, and CB_2_R–GPR55 complexes have been described in the mammalian, including human, brain (e.g., Kargl et al. 2012; Moreno et al. 2014; Solymosi and Köfalvi, 2017; Menéndez-Pérez et al. 2024). Accordingly, robust interpretation of cannabinoid receptor pharmacology will ultimately require the combined use of selective ligands and complementary genetic approaches.

Together, these considerations highlight the need for caution when attributing metabolic effects to individual cannabinoid receptors in brain preparations and emphasize the importance of systematic, cell-type-resolved analyses of receptor expression, including splice variants, across species.

Our final concern is about the antitumoral activity of GPR55 blockade (Vaccani et al. 2005; Singh et al. 2016; Lah et al. 2022), because Δ^9^-THC was also reported to reduce glioblastoma cell proliferation and decrease Ki67 immunostaining following intracranial administration (Guzmán et al. 2006). Previous studies have also shown that Δ^9^-THC and its potent analogue HU210 stimulate glucose uptake and oxidative metabolism in C6 glioma cells (Sánchez et al. 1997), which is not fully aligned with our findings.

However, we only assessed LPI rather than Δ^9^-THC effects on oxphos or lactate release, to avoid confounding data. These and related findings call for further investigation to clarify whether cannabinoid agonists and antagonists, either selective or non-selective, represent meaningful adjuvant therapies in glioblastoma. The evaluation of the safety of medical cannabis in the palliative treatment of cancer cachexia and chemotherapy-induced nausea is also warranted, as it can inadvertently interfere with cancer treatment.

## Supplementary Information

Supplementary Material 1.

## Data Availability

The raw data that support the findings of this study are available from the corresponding author, upon request.

## Abbreviations

[^18^F]FDG PET: [^18^F] Fluorodeoxyglucose positron emission tomography
[¹H]-NMR: Proton nuclear magnetic resonance
[³H]DG: 2-[^3^H] Deoxy-D-glucos
2-AG: 2-arachidonoyl-glycerol
3Rs: Replacement, Refinement and Reduction of Animals in Research
ARRIVE: Animals in Research: Reporting In Vivo Experiments
CB_1_R and CB_2_R: Cannabinoid receptors CB_1_ and CB_2_
CBD: Cannabidiol
*Cnr1*: CB_1_R gene
DMSO: Dimethylsulfoxide
ECS: Endocannabinoid system
EDTA: Ethylenediaminetetraacetic acid
FELASA: Federation for Laboratory Animal Science Associations
GAPDH: Glyceraldehyde-3-phosphate dehydrogenase
GPCR: G protein-coupled receptor
*Gpr55, *GPR55: G protein-coupled receptor 55 (gene)
HCAR1: Hydroxycarboxylic acid receptor 1 or GPR81
HEPES: 4-(2-hydroxyethyl)-1-piperazineethanesulfonic acid
LPI: L-α-lysophosphatidylinositol
mtCB_1_Rs: mitochondrial CB1Rs
PEA: Palmitoylethanolamide
Δ⁹-THC: Δ⁹-tetrahydrocannabinol
