# Supplementary material for: Astrocytic GPR55 receptors promote glycolysis
Source: J Cannabis Res. 2026 Apr 9;8:51. doi: 10.1186/s42238-026-00407-x (PMC13067723; doi:10.1186/s42238-026-00407-x)
Supplement: Supplementary file 1 — Supplementary Material 1. [file 42238_2026_407_MOESM1_ESM.pdf]

Supplementary Data

Astrocytic GPR55 Receptors Promote Glycolysis

Cândida Dias [1,2], Erik Keimpema [3], Rui A. Carvalho [4,5], Daniela Madeira [1], Liliana Dias [1], Ana Ledo [1,2], João Laranjinha [1,2], Rodrigo A. Cunha [1,6], Paula Agostinho [1,6], Tibor Harkány [3,7], Attila Köfalvi [1,8*]

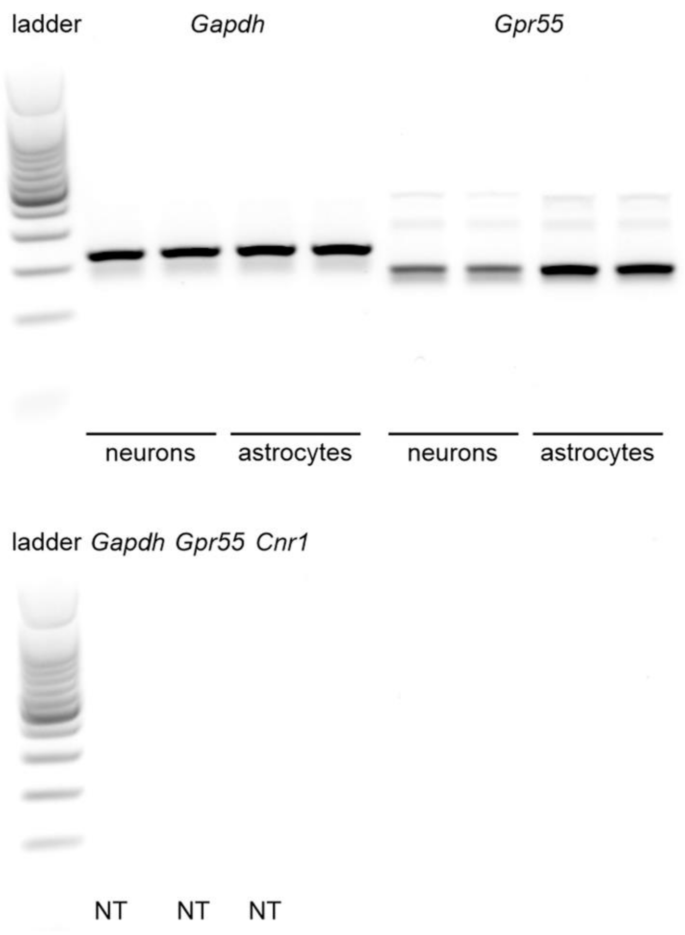

Supplementary Fig. 1 Representative and annotated uncropped gel of the endpoint PCR products after 40 cycles and serve to verify the specificity of the primers and the expected amplicon size. The gel bands represent amplicon verification, confirming that the primers produced a single PCR product of the expected size without unspecific products or primer – dimers. Because they were obtained after 40 cycles, the bands appear strong and visible, but their intensity is not quantitative and does not reflect expression levels. Actual quantification was derived from cycle threshold values measured during the exponential amplification phase and presented in the bar graphs in Fig. 2. NT: "no-template" (*i.e.* negative control).
